# The Biochemical and Pharmacological Properties of Ozone: The Smell of Protection in Acute and Chronic Diseases

**DOI:** 10.3390/ijms20030634

**Published:** 2019-02-01

**Authors:** Rosaria Di Mauro, Giuseppina Cantarella, Renato Bernardini, Michelino Di Rosa, Ignazio Barbagallo, Alfio Distefano, Lucia Longhitano, Nunzio Vicario, Daniela Nicolosi, Giacomo Lazzarino, Daniele Tibullo, Maria Eugenia Gulino, Mariarita Spampinato, Roberto Avola, Giovanni Li Volti

**Affiliations:** 1 Department of Biomedical and Biotechnological Sciences, University of Catania, Via S. Sofia 97, 95125 Catania, Italy; rosariadimauro@tiscali.it (R.D.M.); gcantare@unict.it (G.C.); renato.bernardini@gmail.com (R.B.); chitotriosidase@gmail.com (M.D.R.); distalfio@gmail.com (A.D.); lucialonghitano@hotmail.it (L.L.); vicarionunzio@gmail.com (N.V.); danielanicolosi03@gmail.com (D.N.); d.tibullo@unict.it (D.T.); mariaeugeniagulino@hotmail.it (M.E.G.); mariaritaspampinato93@gmail.com (M.S.); ravola@unict.it (R.A.); 2 Department of Drug Sciences, University of Catania, Viale Andrea Doria, 6, 95125 Catania, Italy; ignazio.barbagallo@unict.it; 3 Institute of Biochemistry and Clinical Biochemistry, Catholic University of Rome, 00168 Rome, Italy; giacomo.lazzarino@unicatt.it; 4 Fondazione Policlinico Universitario A. Gemelli IRCCS, 00168 Rome, Italy; 5 EuroMediterranean Institute of Science and Technology, 90139 Palermo, Italy

**Keywords:** ozone, medical gas, organ protection, oxidative stress, inflammation

## Abstract

Ozone therapy has been widely used in everyday clinical practice over the last few years, leading to significant clinical results in the treatment of herniated discs and pain management. Nevertheless, further studies have demonstrated its potential efficacy and safety under other clinical and experimental conditions. However, some of these studies showed controversial results regarding the safety and efficacy of ozone therapy, thus mining its potential use in an everyday clinical practice. To this regard, it should be considered that extensive literature review reported the use of ozone in a significant different dose range and with different delivery systems. The aim of the present review is to describe the various pharmacological effects of ozone in different organs and clinical conditions and to provide possible biochemical and molecular insights for ozone biological properties, thus providing a possible explanation for various controversial clinical outcomes described in the scientific literature.

## 1. Introduction

The first identification of ozone as a distinct chemical compound was done by Schönbein at the local Naturforschung Gesellschaft in Basel showing that following electrolysis, water emanated an odour at the catode defined as “the odour of electrical matter” which was later on defined by Schönbein as “ozone”, from the Greek *ozein* (odorant). Furthermore, he also suggested that ozone, besides being an oxidant, may also be exploited as a strong disinfectant. This hypothesis was further validated in the late 19th century, when several reports showed the oxidation of a large number of organic compounds and the inactivation of bacterial contaminants in sewage following ozone exposure. Therefore, ozone was also proposed as an alternative treatment to water chlorination. In 1845, de la Rive and Marignac proposed that ozone is an allotropic form of oxygen. Only in the 20th century, Mulliken and Dewar clarified the ozone molecular structure, proposing the acute-angled model [1]. These studies demonstrated that ozone has a molecular weight of 48 with a solubility in water about ten-fold higher than oxygen (49 mL in 100 mL, 0.02 M, at 0 °C) and in its gaseous form spontaneously decomposes with a half-life of 40 min at 20 °C. 

Ozone is present as the main photochemical component of polluted air and leads to a dose-dependent oxidative stress because of its ability to produce free radicals deriving from the lipoperoxidation of cell membranes, protein oxidation, enzymatic inactivation, the destruction of DNA, and cell apoptosis [2]. However, ozone therapy has been widely used in everyday clinical practice over the last few years, leading to significant clinical results in the treatment of herniated discs and pain management [3]. Furthermore, additional clinical evidence has suggested that ozone therapy may play a major role for the treatment of other conditions such as vascular and immune diseases [4,5].

The aim of the present review is to describe the various pharmacological effects of ozone in different organs and clinical conditions and to provide possible biochemical and molecular insights for ozone biological properties, thus providing a possible explanation for various controversial clinical outcomes described in the scientific literature.

### General Aspects of Ozone Mechanisms of Action and Effects on Cell Metabolism

The observed pharmacological effects of ozone underlie its chemical and biochemical properties. The pharmacological properties of ozone are depending on ozone being a triatomic oxygen molecule, reacting with organic compounds containing double bonds and adding the three oxygen atoms to the unsaturated bond with the formation of ozonides. This reaction is of great importance, since ozone causes the split of the double bonds with a reaction called ozonolysis. In an aqueous medium (i.e., blood), the ozonides are immediately transformed into stable hydroperoxides having the ability to release oxygen when the pH increases, as it occurs in protonic environments. Such a physical-chemical characteristic is typical of degenerative processes and/or ischemic conditions. The lipoperoxides derived from the breaking of a chain of ozonides lose the hydrophobicity characteristic of lipids and become soluble in water since they are short-chain lipid compounds. It is generally understood that the toxic effects of ozone are mediated through free radical reactions, although ozone is not a radical species per se [6]. Two different mechanisms may be advocated to explain the ozone-derived radical formation: a direct mechanism involving the oxidation of biomolecules to give classical radical species (hydroxyl radical) and a mechanism involving the radical-dependent production of cytotoxic, nonradical species (aldehydes) [7].

However, in order to fully elucidate the biochemical basis underlying the pharmacological effects of ozone, it is important to illustrate its effects on various coenzymes which are responsible for ozone cell metabolism regulation. To this regard, one of the main effects of ozone is the acceleration of glycolysis; as is known, in fact, a fundamental condition for guaranteeing the continuity of this process is the constant reoxidation of NADH as it occurs following ozone exposure. As far as concerning protein metabolism, ozone intervenes mainly due to its remarkable affinity towards sulfhydryl groups such that it occurs when reacting with glutathione. Similarly, ozone reacts with essential amino acids such as methionine, tryptophan, and other amino acids containing sulfur (i.e., cysteine). In this case, the amino acids are protected from ozone inactivation by two reactions that prevent their degradation: first the oxidation of glutathione and then the oxidation of the coenzymes NADH and NADPH, key reactions in the biochemical mechanism of ozone. Finally, ozone reacts directly with unsaturated fatty acids, which have a double carbon bond and are therefore available for an oxidative reaction, leading to the formation of peroxides following hydrolytic cleavage of the lipid chains. The lipid chains are thus fragmented with a loss of their hydrophobic character and are transformed into hydrophilic components.

## 2. Ozone Therapy and Pulmonary Diseases

Previous studies showed that ozone exhibits controversial effects in pulmonary disease. In particular, Leroy et al. [8] showed that the exposure of human subjects to ozone (200 ppb) results in a significant increase in the expression of various genes involved in the wound healing process (i.e., osteopontin). The authors included in their study nineteen subjects, with and without asthma, exposed to clean air (0 ppb), low (100 ppb), and high (200 ppb) ambient levels of ozone for 4 h on three separate occasions in a climate-controlled chamber. Successively, the subjects recruited for the study were treated with bronchoscopy with bronchoalveolar lavage (BAL) 20 h after the end of exposure. In order to further explain the controversial effects of ozone in pulmonary diseases, we further analyzed the authors’ datasets from the NCBI GEO (Available online: http://www.ncbi.nlm.nih.gov/geo/) databank under accession number GSE58682 (GPL6244), in order to delineate a gene expression profile able to determine an analysis of gene ontology. In particular, we focused our attention to the data of healthy subjects and compared 18 subjects exposed to clean air (0 ppb) vs. 19 subjects to high (200 ppb) ambient levels of ozone. By restricting the threshold level of significance to *p* < 0.01 and the log10 fold change to 0.7 for the upregulated genes and −0.7 for the downregulated, we identified 29 upregulated and 309 downregulated genes in subjects exposed to 200 ppb of ozone, compared to the subjects exposed to clean air. 

**Figure 1:**
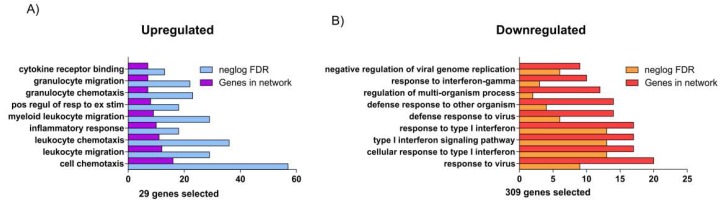
The Gene Ontology analysis: (**A**) The dataset analysis showed that the 29 upregulated genes were involved in several immunological processes. The most significant process was Cell Chemotaxis (the negative Log of FDR (ngLogFDR) was 58, and the genes involved (GI) were 18). (**B**) The 309 downregulated genes identified during the GSE58682 analysis belong to the response to type I interferon (FDR = 14, GI = 16). Interestingly, the ability of antiviral response in subjects exposed to the ozone was significantly impaired.

**Figure 2:**
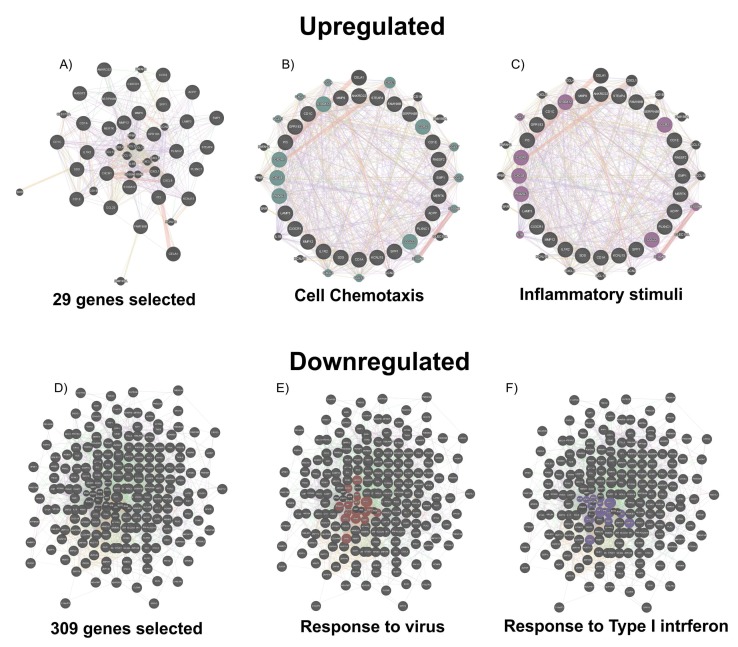
The GeneMania network representation of the 29 upregulated and 309 downregulated genes: (**A**) The GO analysis of 29 genes involved during the exposition to a high ppb of ozone expresses (**B**) the cell chemotaxis and (**C**) inflammatory stimuli as biological processes. (**D**) The 309 downregulated genes in subjects exposed to a high ppb of ozone belong to the biological processes as a response to (**E**) the virus and to (**F**) Type I interferon.

Our Gene Ontology (GO) analysis was performed using the web utility GeneMANIA (Available online: http://genemania.org/) [9] and the GHATER (Gene Annotation Tool to Help Explain Relationships) (Available online: http://changlab.uth.tmc.edu/gather/) [10], allowing us to assemble all the available interaction data in the dataset by creating large networks, which captures the current knowledge on the functional modularity and interconnectivity of genes in a cell. During our analysis, we identified 29 upregulated and 309 downregulated genes significantly expressed in the BAL of healthy subjects exposed to 200 ppb of ozone for 4 h compared to exposed only to clean air. The GO analysis showed surprising results. Indeed, following ozone exposure, the alveolar parenchyma responds with the activation of genes involved in immuno-activation. Processes such as chemotaxis (cell chemotaxis, leucocyte chemotaxis, and granulocyte chemotaxis), responses to cytokines (cytokine receptor binding), and inflammation were significantly triggered (Figure 1A and Figure 2A–C). 

**Figure 3:**
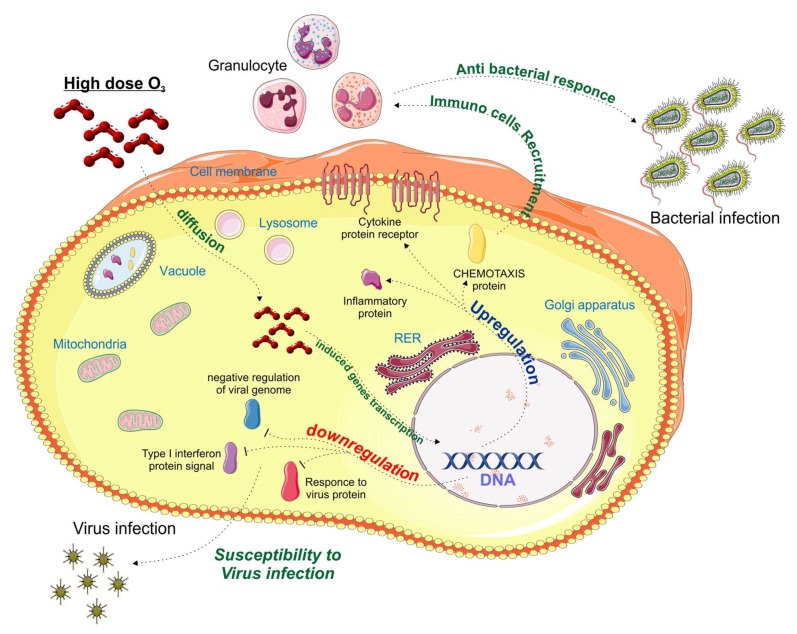
High doses of ozone induce the gene transcription of the pro-inflammatory cytokine, its receptor, and inflammatory proteins. At the same time, we assist at a negative regulation of type 1 Interferon and the response to viral infections pathways.

On the other hand, the downregulation of several genes involved in the antiviral response and in the regulation of type I interferons could, in the long term, predispose to a susceptibility to opportunistic infections (Figure 1B, Figure 2D–F, and Figure 3). Our findings are consistent with previous reports [11], showing that ozone exposure reduces the ability of mice to survive a *K. pneumoniae* infection by reducing the phagocytic ability of alveolar macrophages.

## 3. Ozone Therapy and CNS

**Figure 4:**
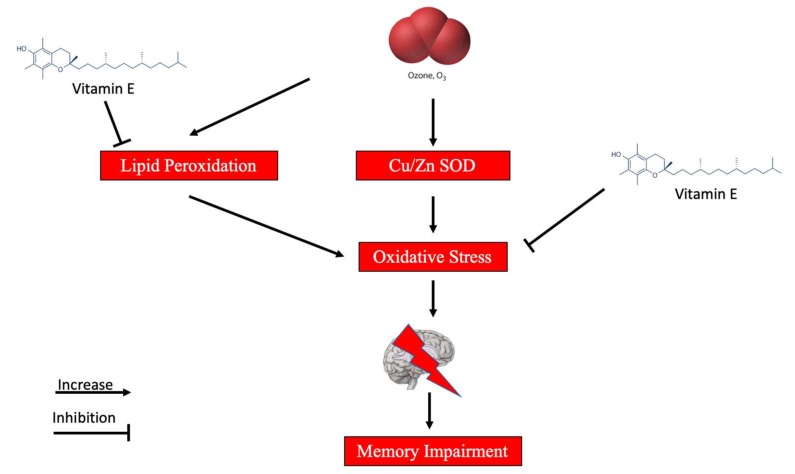
A schematic representation of the possible mechanisms of action of ozone on the CNS.

Recent studies have reported that the nervous system is affected by ozone exposure. In particular, an increase in slow wave sleep time and a decrease in the total time of paradoxical sleep have been found in cats exposed to 1 ppm (parts per million) of O_3_ [12]. Similarly, changes in mental performance, fatigue, headaches, and lethargy have been referred to in humans exposed to this gas [13]. To this regard, several reports suggest that ozone leads to increased oxidative stress in the central nervous system (CNS), which in turns leads to significant brain dysfunction as measured by cognitive and motor activity impairment (Figure 4). In particular, Rivas-Arancibia et al. showed that rats exposed to various ozone concentrations (0, 0.1, 0.2, 0.5, or 1 ppm) exhibited deterioration of their long-term memory in the passive avoidance test and decreased motor activity [14]. Furthermore, the authors suggested that such functional impairment was also accompanied by increased Cu/Zn SOD levels, thus suggesting that ozone exposure affects long-term memory possibly in association with oxidative stress (Figure 4). Consistently with this hypothesis, Guerrero et al. [15] demonstrated that vitamin E (50 mg/Kg) prevented ozone exposure-mediated lipid peroxidation and mitigated brain dysfunction as measured by short- and long-term memory improvement in a passive avoidance task. Consistently, Avila-Costa showed that ozone inhalation led also to morphological changes in the brain with particular regards to the hippocampus CA1 region [16]. In particular, the authors showed that ozone exposure (1 ppm) resulted in a significant reduction in spine density in the pyramidal neurons of the hippocampus and that this may account for the previous described memory impairment. Similarly, other areas of the CNS are particularly involved in ozone-mediated toxicity, including the central respiratory areas (i.e., nucleus tractus solitaries and ventrolateral medulla). Ozone exposure leads to increased oxidative stress in these regions and, as a result of such oxidative insult, astroglial cells increased the vascular endothelial growth factor (VEGF) expression as a potential protective mechanism persisting following the ozone exposure cessation (Figure 4). Consistently, Gackière et al. [17] showed that the exposure of rats to various concentrations of ozone (0.5 to 2 ppm) for a time interval included between 1.5 and 120 h determined a significant time- and dose-dependent neuronal activation in the dorsolateral regions of the nucleus tractus solitarius. Interestingly, the authors also showed neuronal activation in other interconnected central structures, including the caudal ventrolateral medulla, the parabrachial nucleus, the central nucleus of the amygdala, the bed nucleus of the stria terminalis, and the paraventricular hypothalamic nucleus. The existence of a possible lung–brain axis in ozone-mediated cytotoxicity was further supported by Mumaw CL et al. [18], demonstrating that inhaled ozone increased microglial activation via the MAC1 receptor independently from circulating proinflammatory cytokine release and in an age-dependent manner. 

On the other hand, it should be reported that there exists strong scientific and clinical evidence supporting the beneficial effects of ozone for the treatment of some neurological disorders. However, such results are not contradicting previous mentioned studies since in most of the cases, ozone was administered locally and since animals and/or patients were not exposed to ozone by inhalation. The first evidence of the beneficial effects of ozone in neurological disorders refers to the successful treatment of a case of intractable pain in the head and face associated with pathological changes in the optic thalamus [19]. Since then, ozone has been used to treat neuropathic pain allodynia, and hyperalgesia is defined as an increased sensitivity to normally painful stimuli [20,21]. The use of ozone for the treatment of neuropathic pain has been proposed for years based on its antioxidant and anti-inflammatory potential; however the clear molecular cut underlying the analgesic effect of this gas on such clinical conditions has not been fully elucidated. Interestingly, Fuccio et al. [22] showed that a subcutaneous injection of ozone (90 or 180 μg/kg) decreased mechanical allodynia and normalized the mRNA caspase-1, caspase-12, and caspase-8 gene levels in an animal model of the neuropathic model. Interestingly, a subcutaneous injection of ozone translated also in significant pharmacological effects in the orbito-frontal cortex, normalizing the expression of pro-inflammatory caspases and reducing IL-1β staining. Such results were extensively confirmed in a clinical setting and provided significant evidence for the efficacy and safety of this treatment in various cohorts of patients. In particular, a multicenter randomized, doubled-blind, simulated therapy-controlled trial in patients suffering with acute low back pain showed that intramuscular lumbar paravertebral injections of an oxygen–ozone mixture was safe and effective in relieving pain and reduced both disability and the intake of analgesic drugs.

Other beneficial effects of ozone therapy have been associated with a significant improvement in cognitive functions and mood status. In particular, Coppola et al. [23] empirically found that geriatric patients with peripheral occlusive disease and undergoing the reinfusion of autologous blood exposed to ozone exhibited significant improvements in mood tones within hours with no relation to the general clinical condition. Furthermore, the authors evidenced that such an improvement was also accompanied by a significant increase of the brain-derived neurotrophic factor. These results were further confirmed in an experimental model of stroke demonstrating a significant reduction of the neuronal injury and radical formation [24]. Finally, rectal insufflation of ozone exhibited a significant anti-inflammatory effect in an experimental model of multiple sclerosis demonstrating a pharmacological efficacy comparable to methyl-prednisolone [25].

## 4. Ozone Therapy and Skin Diseases

Because of its safety, ozone therapy has been used for many years as a method ancillary to basic treatment, especially in those cases in which traditional treatment methods do not give satisfactory results, e.g., skin loss in non-healing wounds, ulcers, pressure sores, fistulae, etc. [26,27]. 

The first evidence dealing with the beneficial effects of ozone in skin disease was provided by Shpektorova [28] in 1964. Consistently, Białoszewski et al. [29] treated ozone patients with heavy, chronic, antibiotic-resistant septic complications after trauma, surgical procedures, and secondary skin infections, showing that in the wounds of the all experienced patients, the inhibition of septic processes and wound healing was much faster than normal. A secondary result which should be taken into due account is that this method also lowers the cost of antibiotic therapy.

**Figure 5:**
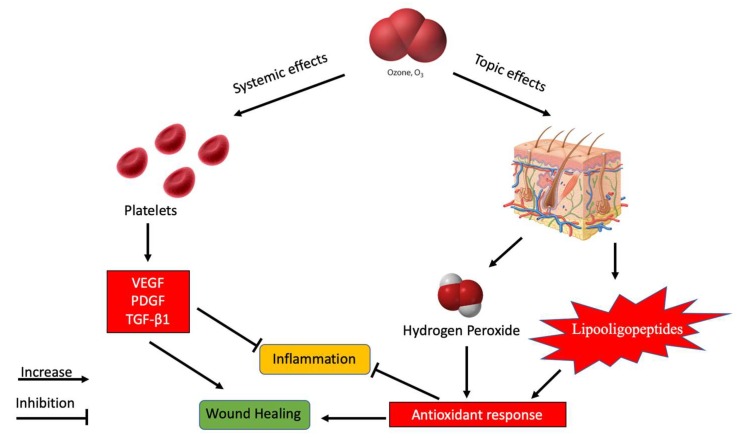
A schematic representation of the possible mechanisms of action of ozone on skin diseases.

In order to fully elucidate the mechanisms underlying the pharmacological effects of ozone in the skin, some important anatomical and functional aspects should be taken into due account. In particular, ozone has a low penetration potential into cutaneous tissues since it rapidly reacts with polyunsaturated fatty acids and water of the stratum corneum leading to the formation of ROS and lipooligopeptides (LOP) which in turn are readily scavenged by skin antioxidants and/or partially absorbed via the venous and lymphatic capillaries (Figure 5). The safety of non-inhaling ozone therapy is also substantiated by clinical evidence obtained with healthy volunteers following exposure to a mixture of oxygen and ozone using a closed cabin allowing a temporary exposure of the skin to concentrations as low as 0.9 μg mL^−1^ [30]. Interestingly, the authors showed that under such clinical condition, there was a significant increase of pO_2_ and of peroxidation products in the venous plasma with a concomitant feeling of well-being in the next few days. A different approach to exploit the beneficial effect of ozone overcoming the instability of ozone in pharmaceutical preparation is the ozonide. Such preparations are of possible clinical interest since they are stable for 2 years at 4 °C and may be used in the treatment of various infections of cutaneous and mucosal areas. Ozonated oil is currently used topically for wounds repair, anaerobic and herpetic infections, trophic ulcers, burns, cellulitis, abscesses, anal fissures, fistulae, gingivitis, and vulvovaginitis [31]. Consistently with these observations, Matsumoto et al. showed that ozonated oil is effective in almost all enrolled patients in treatment for fistulae and chronic surgical wounds without side effects [32]. Similarly, exposure to ozone significantly reduced the severity of radiodermatitis lesions in patients with cancer [33]. Furthermore, Kim et al. [34] suggested that ozonated oil promotes acceleration in acute cutaneous wound repairs by the increased expression of PDGF, TGF-β1, and VEGF (Figure 5). 

Finally, it was reported that ozone exposure is associated with the activation of transcription factor NF-κB, playing a pivotal role in the inflammatory response regulation and wound healing mechanisms [35,36,37,38]. Furthermore, previous reports showed that significant PDGF and TGF-β1 levels were released from platelets in the heparinized plasma of a limb ischemia patient after ozonation (Figure 5) [31,39]. Consistently, a previous study has shown that hydrogen peroxide (H_2_O_2_) potently induced the VEGF expression in human keratinocytes, which can stimulate wound healing [40]. From these previous studies, the authors hypothesized that ozone might enhance acute cutaneous wound healing, and this could be associated with growth factors such as FGF, PDGF, TGF-β, and VEGF (Figure 5). Similarly, Krkl et al. [41] showed that ozonated olive oil improved neovascularization in rats when it was topically applied on skin flaps via VEGF upregulation. In addition, ozonated oil reduced doxorubicine-induced skin necrosis and injury as measured by a significant reduction of TNF-α, SOD, GSH-Px, and IL-1β [42] and by the enhanced flap viability in a rat model [43].

## 5. Ozone Therapy and CVD

Cardiovascular diseases (CVD) are a major factor in mortality rates around the world [44]. Increasing evidence has highlighted the roles of oxidative stress and inflammation in the promotion of CVD [45], leading to impaired endothelial function, a process which promotes atherosclerotic lesion or fatty streak formation (foam cells) associated with ischemic heart disease, stroke, and major debilitating events [46,47]. In this context, ozone therapy may represent a possible approach for correcting oxidative stress and thus improve the prognosis of many patients [48,49].

**Figure 6:**
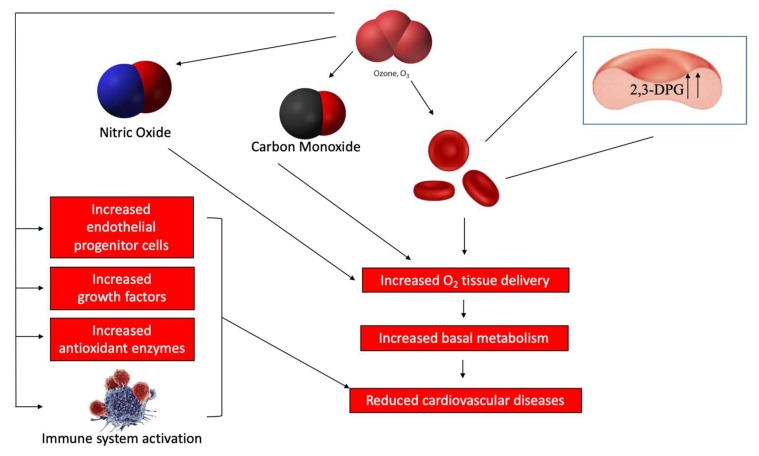
A schematic representation of the possible mechanisms of action of ozone in skin diseases.

Ozone, by acting on multiple targets, is indirectly involved in recovering functional activities impaired by chronic disease. On the basis of the mechanisms of action, ozone therapy induces a biological response including the improvement of blood circulation and oxygen delivery to ischemic tissue as a result of the concerted effect of NO and CO, an increase in intraerythrocytic 2,3-DPG level (Figure 6) [50,51], an increase in reduced glutathione, an enhancement in basal metabolism through improved oxygen delivery, an upregulation of cellular antioxidant enzymes, the induction of HO-1 and HSP-70 [52,53], the induction of a mild activation of the immune system, and the increased release of growth factors in the absence of acute or late adverse effects (Figure 6). Therefore, these properties suggest that ozone properties may be exploited in the treatment of CVD. In fact, several in vitro and in vivo studies, as well as some clinical trials, have shown a positive effect in cardiovascular disorders such as coronary artery disease (CAD), chronic heart failure (CHF), myocardial infarction, and peripheral artery diseases. To this regard, a clinical trial conducted by Martinez-Sanchez et al. demonstrated that integrative therapy with ozone resulted in a beneficial treatment of CAD and its complications [54]. In particular, this study showed that the beneficial effect of ozone therapy was related to a reduction of SOD and catalase activities, advanced oxidation protein products, malondialdehyde, peroxidation potential, and total hydroperoxides with a concomitant increase in GSH levels and the ferric reducing ability of plasma, leading to a low production of O_2_^•−^ and reduced oxidative stress. Similarly, several studies showed that ozone therapy in patients with ischemic heart disease or had suffered an acute myocardial infarction (AMI) resulted in favorable effects in terms of pain and prognosis. Furthermore, in a case report of a 76-year-old patient suffering from a myocardial infarction with parkinsonism, hypertension, chronic renal disease, and dyslipidemia as comorbidities, the authors showed that large autologous ozonized blood infusions for 18 months resulted in an improved myocardial contractility and in the protection against the risk of subsequent recurrences of AMI (Ozone Therapy 2017; volume 2:6745). 

In order to elucidate the possible pharmacological mechanisms of ozone therapy, in a previous study, the authors exposed rats to an oxygen/ozone mixture prior to an acute ischemia/reperfusion injury of the myocardium, demonstrating a significant decrease of the myocardial infarcted area associated with an increased recruitment of endothelial progenitor cells (EPCs) within the myocardium [55] (Figure 6). Furthermore, in this study, the protective effect of oxygen/ozone was closely related to the increase in cardiac eNOS expression and activity. 

In addition, Barone et al. demonstrated that ozone therapy reduced restenosis 30 days following percutaneous transluminal coronary angioplasty with metal stent implantations in pigs [56]. The authors suggested that ozone autohemotransfusion upregulated the innate detoxifying and antioxidant system (i.e., thioredoxins), preventing post-stenting neointima hyperplasia and an inflammatory reaction, thus supporting successive re-endothelization which may be dependent on the increased glycolysis rate, stimulation of NO synthesis, and induction of growth factors [57,58]. Moreover, several studies demonstrated that in patients with occlusive peripheral arterial disease (PAD), the reinfusion of autologous blood after ozonation lead to correcting the altered hemostatic-hemorheological parameters improving blood flow and the release of O_2_ from hemoglobin into the tissues [59,60,61]. It was also demonstrated that autohemotransfusion with ozonized blood per se does not significantly influence the fibrinolytic balance [62]. However, ozone therapy was more effective than prostacyclin in treating skin lesions in PAD patients, resulting also in a significant improvement of patient general conditions [63]. Furthermore, more studies showed that in patients with peripheral occlusive atherosclerotic disease (POAD), the ozone therapy improved the symptoms of intermittent claudication [64,65]. 

## 6. Conclusions

As far as demonstrated for other medical gases used in the clinical practice (i.e., O_2_, NO, and CO), ozone can be either toxic or safe as a real drug, depending upon its dosage, length of exposure, and the antioxidant capacity of the tissue exposed. Based on the pharmacological implications and clinical evidences, it can be concluded that the use of medical ozone can be advantageous in the treatment of various diseases. The fact that such diseases may have different connotations between them makes reason for its controversial effects. However, it is essential that precise guidelines are observed in therapeutic applications and that the concentrations to which this mixture is used are within a non-toxic range. To this regard, future studies are now warranted in order to establish a valid confirmation and a more exhaustive explanation underlying the molecular pharmacological and biochemical effects of ozone.
